# Contamination With Fumonisin B and Deoxynivalenol Is a Threat to Egg Safety and Contributes to Gizzard Ulcerations of Newborn Chickens

**DOI:** 10.3389/fmicb.2021.676671

**Published:** 2021-07-12

**Authors:** Yihui Wang, Hongkun Quan, Xiaohui Li, Qiang Li, Md Atiqul Haque, Qin Shi, Qiang Fu, Cheng He

**Affiliations:** ^1^College of Life Science and Engineering, Foshan University, Foshan, China; ^2^Key Lab of Animal Epidemiology and Zoonoses, College of Veterinary Medicine, China Agricultural University, Beijing, China

**Keywords:** fumonisin B, deoxynivalenol, residual, breeder eggs, gastric ulceration, newborn chicken

## Abstract

Fumonisin B (FB) and other fumonisins, deoxynivalenol (DON), and zearalenone (ZEN) are mycotoxins (secondary metabolites of fungi) present at high levels of contamination in poultry diets and threatening the sustainability of the poultry industry and egg safety for consumers. However, residual mycotoxins in breeder eggs and their effects on chicken progeny and gizzard ulcerations remain unclear. To unveil mycotoxin contaminations from daily diets to breeder eggs, 293 poultry feed samples were collected from three large-scale poultry provinces across Northern China to Southern China. Average levels of 1,628 ± 4.36 μg/kg of FB_1_, 593 ± 11.16 μg/kg of DON, 69 ± 9.21 μg/kg of ZEN, 52 ± 7.33 μg/kg of OTA, and 24 ± 5.85 μg/kg of AFB_1_ were found in feedstuffs and poultry diets using commercial ELISA kits. In terms of residual mycotoxins in breeder eggs, FB_1_ and DON contaminations dominated residues in egg albumen and yolk samples. Out of 221 breeder eggs, the average residual of FB_1_ in albumen were 320.6 ± 10.12 μg/kg (Hebei), 420.2 ± 10.98 μg/kg (Guangdong), and 549.4 ± 10.27 (Guangxi). Moreover, higher residual of DONs were determined in Guangdong and Guangxi provinces compared to Hebei province. ZEN, ochratoxins A (OTA), and aflatoxin B_1_ (AFB_1_) contamination at low levels were found in the above samples collected from afronmentioned three provinces. Based on residual mycotoxins in breeder eggs, SPF embryonated eggs aged 11 days were inoculated into albumen with different doses of FB_1_, FB_2_ or DON, or a combination of FB_1_ and DON, or a combination of FB_1_ with FB_2_ and FB_3_. A lower hatching rate was observed in the chicken progenies with the combination of 24 μg of FB_1_ and 0.1 μg of DON compared to other treatments. Moreover, typical gizzard ulcerations with hemorrhagic lungs were observed in the progeny of breeder eggs post-inoculation of 24 μg of FB_1_ and synergetic inoculation of FB_1_ and DON. Finally, residual FB mycotoxins were detected in the gizzards and in the lungs of the progenies. Based on the above evidence, feed-borne FB_1_ and DON are dominant mycotoxins in breeder eggs and threatening food security using breeder eggs as a Trojan horse. More importantly, the residual of FB_1_ alone and in combination with of DON contamination are associated with low hatching rate and gizzard ulcerations in chicken progenies, hampering sustainable development perspectives of the poultry industry.

## Introduction

Avian gizzard ulceration has been documented frequently in the poultry industry, contributing to consistent diarrhea, poor feed conversion, and low economic benefits. Gizzard ulceration is associated with congenital factors, starvation, feed structure, nutritional deficiencies, toxic substances, infections, and microbial colonization; however, the potential mechanism remains unclear, threatening the sustainability of the poultry industry (Atiqul Haque et al., 2020).

At present, the hatching rate is less than 86.5–90.8% during the summer season in Chinese poultry farms, equal to an average of 9.5–13.5% hatching reduction in comparison with the cold season. More importantly, gizzard ulcerations are prevalent in newly hatched chickens among fast-growing broilers, slow-growing broilers (yellow broilers), and ducklings. Consequently, newborn chickens and ducklings are suffering from an average 15% mortality due to severe diarrhea and maldigestion in the first week. Unfortunately, no practical control measure is available due to elusive pathogenesis. In a previous study, *adenovirus* serotype 4 was identified from hemorrhagic ventricular gastric lesions in broiler flocks in South Korea, Japan, and Italy (Manarolla et al., 2009). Subsequently, *Clostridium perfringens (C. perfringens)* was isolated from gizzard ulceration in commercial chickens. *C. perfringens* is more than an opportunistic pathogen occupying a new niche following the appearance of gizzard lesions. *Bacillus cereus* (*B.cereus*), as a primary or permanent latent infection, induced gizzard ulceration and lung inflammation in SPF chickens and aggravated susceptibility to *avian influenza virus* H9N2 (Zhang et al., 2019). Chickens’ exposure to *B. cereus* intensifies gizzard ulceration and pneumonia after chlamydial infection, leading to respiratory stress and breathing dysfunction (Zuo et al., 2020).

Recently, increasing levels of mycotoxins have been found in poultry and livestock diets. Mycotoxin contamination is particularly present in the hot season with high humidity. In previous reports, a study in Kenya showed that the final flour was less contaminated, while mycotoxins of deoxynivalenol (DON) and zearalenone (ZEN) were detected on the surface of the granules at high levels while temperature and time can affect the mycotoxin content of the final product (Agriopoulou et al., 2020). Dysfunctions of the liver and kidney were exacerbated in the layers after intravenous injection of ZEN, DON, and fumonisin B_1_ (FB_1_) (Dassi et al., 2018). Numerous reports have documented the association between feed-borne mycotoxin and gastrointestinal tract (GIT) issues in broilers and growing pigs. Mycotoxins reduce broiler feed intake by 12% and weight gain by 14%, with aflatoxin B_1_ (AFB_1_), ochratoxins A (OTA), and deoxynivalenol (DON) having the greatest effect on these parameters. The GIT is the initial site for interaction of ingested mycotoxins in the animal, and mycotoxins have a negative effect on the viability of the intestinal cells (Broom, 2015). After feeding with FB_1_ contaminated diets, villus height and crypt depth of the ileum were significantly reduced in broilers, and a higher percentage of birds developed subclinical necrotic enteritis in the groups fed the FB_1_-contaminated feed as compared to the control group, after the *C. perfringens* challenge (Antonissen et al., 2015).

Generally, egg albumen contains about 10.5% protein and 88.5% water, while the yolk only contains 50% water in the eggs of domestic fowl. This difference might be necessary to ensure that eggs are provisioned with adequate levels of nutrients for incubation and embryo development (Vesely et al., 1982). However, no report on correlation between mycotoxin contamination and gizzard ulcerations of chicken progenies has been recorded. Therefore, our hypothesis is that breeder eggs are highly contaminated with mycotoxins *via* the feed-egg chain, and synergistic mycotoxins might contribute to gastric lesions of new hatching chickens during embryonic development. We collected feed samples and breeder eggs from Northern China to Southern China. Afterward, SPF embryonated chickens, aged 11 days, were inoculated artificially with FB_1_, FB_2_ or DON, or a combination of FB_1_ with DON or synergetic inoculation of FB_1_ with FB_2_ and FB_3_. The hatching rate, lesions, and mycotoxin residues were monitored in lungs and gastric system on hatching day.

## Materials and Methods

### Determination of Mycotoxins in Poultry Feed and Breeder Eggs

A cross-sectional survey was carried out to collect feed samples from three provinces in China—Hebei, Guangdong, and Guangxi—in September 2017, representing a large poultry population both in Northern China and in Southern China. A total of 293 feed samples and 221 breeder eggs were collected from the aforementioned poultry farms, namely, 32 corns, 20 soybeans, 19 brans, 19 choline chlorides, 20 feed additives, 95 start diets, and 88 finished diets. As for breeder eggs, 72, 80, and 69 eggs were collected from Hebei, Guangxi and Guangdong in the same poultry farms, respectively.

Regarding determination of mycotoxins, poultry feedstuff and diets were treated and detected using commercial kits (Beacon Analytical Systems Inc., TX, United States). For measurement of the mycotoxin residues in breeder eggs, the protocols were modified as follows. Firstly, the albumen and yolk were separated using a manual egg separator. Afterward, the egg samples were lyophilized for 24 h to prepare powder form (Thermo Fisher Scientific Inc., Shanghai, China) and kept at −80°C until use. Finally, quantities of FB, DON and ZEN were determined using ELISA kits (Beacon Analytical Systems Inc., United States).

### Effect of Mycotoxins on the Hatching Rate and Gizzard Ulceration of Embryonated Chickens

FB_1_, FB_2_, and FB_3_ samples (purity 98%) were purchased from commercial products (Pribolab Pte. Ltd., Singapore, Singapore). SPF embryonated chickens, aged 11 days, were purchased from a commercial company (Boehringer Ingelheim Inc., Beijing, China). The experimental protocols were approved by an Ethical Reviewing Board at China Agricultural University (Approval code: IACUC201700802), based on guidelines from the Institutional Animal Care and Use Committee (IACUC). This follows humane protocols that minimize pain in the animals. All hatching chickens were euthanized at the end of the study in a CO_2_ chamber using 100% CO_2_ at a flow rate of 10–30% of the chamber volume per minute, and the birds were observed for the absence of breathing activities and loss of heartbeat. The CO_2_ flow lasted for at least 1 min after breathing arrest. After confirmation of death, an additional secondary physical euthanasia was carried out before tissue collection as previously described (Chu et al., 2017).

A total of 112 embryonated chickens, aged 11 days, were randomly divided into six groups, namely, four experimental groups with 24 chicken embryos in each group and two control groups with 8 chicken embryos per group. The experimental groups consisted of the FB_1_ group, the FB_2_ group, the FB_1_ + FB_2_ + FB_3_ group, and the FB_1_ + DON group, while each experimental group was divided into three different doses, the high-dose group, the moderate-dose group and the low-dose group, with eight chicken embryos per group. Meanwhile, the control groups included the DON control group and the methanol reagent control group, with eight chicken embryos per group. The chicken embryos received 0.1 μg of DON or 0.1 ml of methanol as the control group. Briefly, FB_1_ samples were prepared by adding 64 mg of the toxin to 10 ml solutions containing methanol:water (1:1 vol/vol). The FB_2_ and FB_3_ samples were prepared by dissolving 8 mg of the toxins in 5 ml solutions with methanol:water (1:1 vol/vol). The FB_1_ + FB_2_ + FB_3_ stock samples included three ratios of toxins (3:1:1 weight ratio), by adding 9.6 mg of FB_1_, 3.2 mg of FB_2_, and 3.2 mg of FB_3_ to 10 ml solutions containing methanol:water (1:1 vol/vol). Afterward, serial dilutions were prepared as described in Table 1. Finally, the FB1 + DON samples were prepared as described above (Henry and Wyatt, 2001).

**TABLE 1:** Experimental designs with mycotoxin alone or synergetic inoculations.

Groups	Toxins	Doses (μg/egg)
1	FB_1_	24
		12
		6
2	FB_2_	48
		24
		12
3	FB_1_ + FB_2_ + FB_3_	24 (14.4 + 4.8 + 4.8)
		12 (7.2 + 2.4 + 2.4)
		6 (3.6 + 1.2 + 1.2)
4	FB_1_ + DON	0.1 + 12
		0.1 + 6
		0.1 + 3
5	DON	0.1
6	CONTROL	NS

*All embryonated eggs were randomly divided into six groups, namely, four experimental groups (each experimental group consisting of the high-dose group, the moderate-dose group, and the low-dose group, eight replicates per group) and two control groups (the DON control group and the methanol group, eight chicken embryos per group). Before inoculation, poorly growing embryonated eggs were eliminated from the experiment.*

Prior to inoculation into the SPF chicken albumen, poorly growing embryonated eggs aged 11 days were eliminated from the experiment (Willems et al., 2014). Before inoculation, the embryonated eggs were sterilized with 1% iodine and 75% alcohol, and then 100 μl preparations per egg were injected into the embryo albumen. Post inoculation, the injected hole was sealed with wax and embryonated eggs were incubated at 37°C and monitored daily for mortality, development, and hatching numbers.

### Determination of Mycotoxins in the Gizzard and Lungs of Newborn Chickens

#### FB Detection

After hatching at day 20, the newborn chickens were euthanized by CO_2_ flowing chamber, as per the above protocol. Post-mortem, the gizzards and lungs of the newborn chicks were collected aseptically, the organs were homogenized and 1 g of tissue was blended with 10 ml of 70% methanol/ultra-water. Subsequently, the samples were centrifugated at 12,000 rpm for 5 min, and 1 ml of the supernatants was collected and mixed with 2 ml of *n*-hexane. Afterward, the samples were centrifuged to discard *n*-hexane at 12,000 rpm for 5 mins. Finally, 100 μl of the lower samples was collected and 900 μl of 70% methanol/ultra-water was added, and the mixture was blended together. FB concentrations were determined using commercial ELISA kits (Beacon Analytical Systems Inc., TX, United States), as described in the protocol.

#### DON Detection

The sample tissues of gizzards and lungs were prepared as per the above procedures, and the pre-treatment was modified as follows. Briefly, 5 g of tissue was homogenized with 25 ml of ultrapure water and then centrifuged at 12,000 rpm for 5 mins. Afterward, 1 ml of the supernatant was added to 2 ml of *n*-hexane and then centrifuged at 12,000 rpm for 5 mins. Lastly, the upper *n*-hexane was removed and 200 μl of the lower sample was blended with 800 μl of ultrapure water, to be used as the final sample. DON concentrations were quantified using commercial ELISA kits (Beacon Analytical Systems Inc., TX, United States).

### Statistical Analysis

Mycotoxin concentrations and lesion scores were statistically analyzed using SPSS 17.0 version to perform a one-way ANOVA with the LSD *post hoc* test on at least three independent replicates. *p*-values <0.05 were considered statistically significant for each test, and when *p* < 0.01, the results were very significant. Hatching rate was statistically analyzed using SPSS 17.0 version to perform a chi-square test with categorical variable. A *p*-value of <0.05 was considered to be a significant difference for each test, and a *p*-value of <0.01 was considered to be very significant.

## Results

### Determination of Mycotoxins in Poultry Feed and Breeder Eggs

A total of 110 feedstuffs and 183 concentrated feed samples were included in the survey. The average concentrations were 1,628 ± 4.4 μg/kg of FB, 593 ± 11.2 μg/kg of DON, 69 ± 9.2 μg/kg of ZEN, 52 ± 7.3 μg/kg of OTA, and 24 ± 5.9 μg/kg of AFT, while the finished diet and fermented soybeans were determined to contain 3,450 ± 2.6 μg/kg and 2,300 ± 6.2 μg/kg of FB, respectively. As for DON contamination, the feedstuffs were found to have high contamination in the corns, brans, and feed additives, amounting to 678 ± 5.23 μg/kg, 813 ± 3.3 μg/kg, and 1200 ± 8.2 μg/kg, respectively. As for the AFT concentration, low contamination of both the feedstuffs and poultry diets were found in comparison with OTA and ZEN levels (Table 2). In the survey, 221 breeder eggs were collected to determine mycotoxin contamination across three provinces. As for average FB residues, 320 ± 10.1 μg/kg, 420 ± 10.9 μg/kg, and 549 ± 10.3 μg/kg were detected in the albumen samples, while 151 ± 9.8 μg/kg, 321 ± 5.1 μg/kg, and 669 ± 8.5 μg/kg were determined in yolk samples in the three provinces. Moreover, 184 ± 9.3 μg/kg, 507 ± 12.43 μg/kg, and 658 ± 2.62 μg/kg of DON concentrations were quantified in the albumen, which were comparable to the contamination levels in the yolk samples. High concentrations of FB and DON were detected in Guangdong and Guangxi provinces, Southern China, compared to Hebei province, except for FB residues in the albumen. Regarding ZEN, OTA, and AFT contaminations, higher residues were found in the albumens collected from Guangdong and Guangxi provinces, compared to those of Hebei province (Table 3).

**TABLE 2:** Average residues of FB, DON, ZEN, OTA, and AFT in feedstuffs and feeds (μg/kg).

Sample	No.	FB	DON	ZEN	OTA	AFT
Corn	32	1,200 ± 4.8 ^b^	678 ± 5.2^b^	67 ± 10.2^a^	77 ± 4.6^b^	35 ± 11.1^b^
Cardamom	20	2,300 ± 6.2^c^	547 ± 12.1^a^	85 ± 7.8^b^	89 ± 4.3^b^	26 ± 8.2^a^
Bran	19	1,500 ± 3.1^a^	813 ± 3.3 ^b^	86 ± 8.3^b^	17 ± 5.5^b^	14 ± 4.9^b^
Choline chloride	19	236 ± 4.4^c^	56 ± 5.0^c^	56 ± 2.1^a^	15 ± 3.4^b^	9 ± 7.3^b^
Feed additives	20	2,145 ± 8.3^c^	1,200 ± 8.2^c^	100 ± 9.5^b^	46 ± 5.2^a^	29 ± 10.1^a^
Start diets	95	567 ± 7.0^c^	430 ± 4.0 ^b^	130 ± 4.3^c^	39 ± 6.3^b^	31 ± 4.6^a^
Finished diets	88	3,450 ± 2.6^c^	426 ± 8.1^b^	80 ± 4.6^a^	78 ± 5.0^b^	25 ± 8.4^a^
Average		1628 ± 4.4	593 ± 11.2	69 ± 9.2	52 ± 7.3	24 ± 5.9

*^*a*^*p* > 0.05 when compared with the average level of FB or DON, or ZEN or OTA, or AFT residue in the same column. ^*b*^*p* < 0.05 when compared with the average level of FB or DON, or ZEN or OTA, or AFT residue in the same column. ^*c*^*p* < 0.01 when compared with the average level of FB or DON, or ZEN or OTA, or AFT residue in the same column. The data were expressed as the mean ± SD.*

**TABLE 3:** Detection of FB, DON, ZEN, OTA, and AFT in the yolk and albumen of breeder eggs.

Sample point	Hebei		Guangdong		Guangxi	
	Albumen	Yolk	Albumen	Yolk	Albumen	Yolk
No.	72	72	80	80	69	69
FB	320 ± 10.1^b^	151 ± 9.8^b^	420 ± 11.0^a^	321 ± 5.1^b^	549 ± 10.3^b^	669 ± 8.5^b^
DON	184 ± 9.3^b^	192 ± 11.7^b^	507 ± 12.4^b^	471 ± 6.3^a^	658 ± 2.6^b^	515 ± 8.0^b^
ZEN	0.2 ± 1.2^a^	12 ± 2.7^a^	0.7 ± 0.8^a^	23 ± 3.7^b^	0.3 ± 0.5^a^	15 ± 6.3^a^
OTA	2.5 ± 1.6^a^	1 ± 0.6^a^	13.3 ± 1.5^b^	9.7 ± 1.3^b^	7.4 ± 1.3^b^	5.4 ± 1.2^a^
AFT	3.5 ± 1.3^a^	0.8 ± 0.3^a^	1 ± 1.9^a^	0.7 ± 1.7^a^	11.3 ± 1.1^b^	6.2 ± 0.9^b^

*^*a*^*p* > 0.05 when compared with the average level of FB or DON, or ZEN or OTA, or AFT residue in the same row. ^*b*^*p* < 0.05 when compared with the average level of FB or DON, or ZEN or OTA, or AFT residue in the same row. The data were expressed as the mean ± SD.*

### Effect of Mycotoxins on Hatching Rate and Gizzard Ulceration of Embryonated Chickens

**FIGURE 1:**
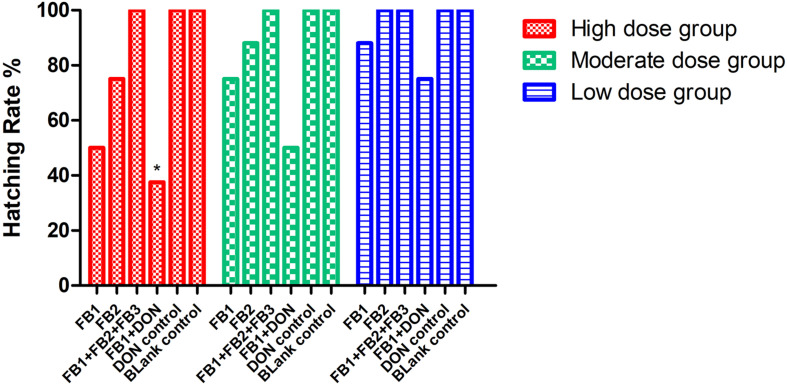
Hatching rate post-inoculation with serial concentrations of FB or DON, or synergetic inoculation. *Indicates *p* < 0.05 when the high FB_1_ + DON group was compared to the high FB_1_ group, the high FB_1_ + FB_2_ + FB_3_ group, and the DON group (*p* = 0.037), while no significant difference was found when the moderate FB_1_ + DON group was compared to other moderate groups and the DON group (*p* = 0.114). Similarly, no statistical difference was found when the low FB_1_ + DON group was compared to other low groups (*p* = 0.427). The data were analyzed by Chi-square test with SPSS as the categorical variable.

**FIGURE 2:**
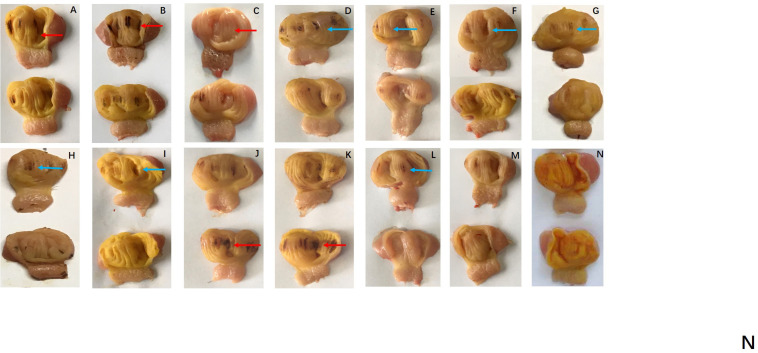
Gizzard ulcerations of chicken progenies post-inoculation with FB or DON, or synergetic inoculation. **(A)** FB_1_ 6 μg; **(B)** FB_1_ 12 μg; **(C)** FB_1_ 24 μg; **(D)** FB_2_ 12 μg; **(E)** FB_2_ 24 μg; **(F)** FB_2_ 48 μg; **(G)** FB_1_ 3.6 μg + FB_2_ 1.2 μg + FB_3_ 1.2 μg; **(H)** FB_1_ 7.2 μg + FB_2_ 2.4 μg + FB_3_ 2.4 μg; **(I)** FB_1_ 14.4 μg + FB_2_ 4.8 μg + FB_3_ 4.8 μg; **(J)** FB_1_ 3 μg + DON 0.1 μg; **(K)** FB_1_ 6 μg + DON 0.1 μg; **(L)** FB_1_ 12 μg + DON 0.1 μg; **(M)** DON 0.1 μg; **(N)** Control group. Severe gastric lesions were marked with a red arrow, and moderate lesions were labeled with a blue arrow. Peeling and shedding of the gizzard membranes were evident both in the high FB1 group and the high FB_1_ + DON group. Additionally, severe hemorrhagic lesions were observed in the above two groups.

**FIGURE 3:**
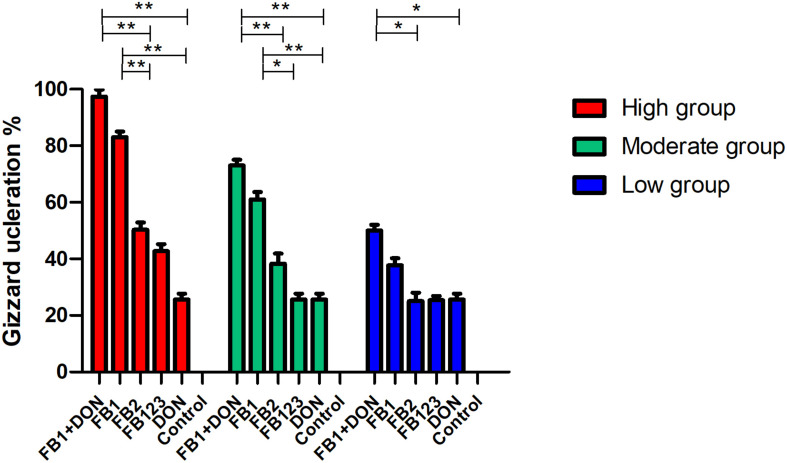
Percentage of gizzard ulceration of chicken progenies post-inoculation with FB_1_ or DON, or synergetic inoculation. Post-mortem, both the high FB_1_ + DON group and the high FB_1_ group developed higher lesions of gizzard ulcerations compared to the high FB_2_ group, or the high FB_1_ + FB_2_ + FB_3_ group or the DON group (*p* < 0.01), while significant difference was found in the moderate FB_1_ + DON group compared to the moderate FB_2_ group, or the moderate FB_1_ + FB_2_ + FB_3_ group or the DON group (*p* < 0.01). As for the moderate FB_1_ group, a statistical difference was found compared to the moderate FB_2_ group, the moderate FB_1_ + FB_2_ + FB_3_ group, and the DON group (*p* < 0.05). The data were expressed as the mean ± SD. *p < 0.05; **p < 0.01.

Regarding hatching rate, 88, 75, and 50% hatching rate were found in the three serial FB_1_ groups (6–24 μg), while 75, 50, and 37.5% hatching rate were found in the serial FB_1_ + DON groups (from 3 to 12 μg FB_1_), indicating a dose-dependent effect. Obviously, a lower hatching rate was observed in the high FB_1_ + DON group compared to the high FB_1_ group, FB_1_ + FB_2_ + FB_3_, the high FB2 group, and the DON group (*p* < 0.05). Moreover, 100, 88, and 75% hatching rate were calculated in the three serial FB_2_ groups (from 12 to 48 μg). However, no embryonic mortality was found in the combination of the FB_1_ + FB_2_ + FB_3_ group and the DON control group (Figure 1). Post-mortem, the gizzard lesions were shown in Figure 2, and a significant increase in gizzard ulcerations was observed in the high FB_1_ + DON group (FB_1_ 12 μg + DON 0.1 μg), the high FB_1_ group (24 μg), the high FB_2_ group (48 μg), and the combination of high FB_1_ with FB_2_ and FB_3_ (FB_1_ 14.4 μg + FB_2_ 4.8 μg + FB_3_ 4.8 μg), amounting to 100, 85, 50, and 45% of the lesions, respectively. In the moderate groups, 75, 63, 38, 25, and 25% of the gastric lesions were found in the mixed group (FB_1_ 6 μg + DON 0.1 μg), the FB_1_ (12 μg) group, the FB_2_ (24 μg) group, the three FB synergetic groups (FB_1_ 7.2 μg + FB_2_ 2.4 μg + FB_3_ 2.4 μg), and the DON group (0.1 μg). Obviously, above 50% gastric lesions were found in the high FB_1_ + DON group, the high FB1 group, the moderate FB_1_ + DON group, the moderate FB1 group, and the low FB_1_ + DON group. A significant increase in gastric lesions was found in both the FB_1_ + DON group and the FB1 group compared to the other groups inoculated with the same dose (*p* < 0.05); the rate of gizzard ulceration of newborn chicken was shown in Figure 3. Regarding the combination of FB_1_ and DON, 37.5% hatching rate and 100% gastric lesions were observed in the high FB_1_ + DON group, indicating that two mycotoxins yielded a synergetic effect on development of the embryonated chickens.

### Determination of Mycotoxins in the Gizzard and Lungs of Newborn Chickens

Using a commercial ELISA kit, gizzard-contaminated FB mycotoxins were determined to have a dose-dependent effect in the FB_1_ + DON group, the FB_1_ group, the FB_2_ group, the three FB combination group, and the DON group. Consequently, 260 ± 27.3, 129 ± 21.0, and 98 ± 10.2 μg/kg of FB were detected in the gizzard tissues in the high FB_1_ + DON group, the moderate FB_1_ + DON group, and the low FB_1_ + DON group, respectively. Obviously, significantly higher FB mycotoxins were found in the high FB_1_ group as compared to the moderate FB_1_ + DON group (*p* < 0.05) and the low FB_1_ + DON group (*p* < 0.01). Moreover, a significant difference was found between the high FB_1_ group and the low FB_1_ group. A similar trend was found among the FB_2_ groups and the FB_1_ + FB_2_ + FB_3_ groups.

On contrary to the mycotoxin residue in the gizzards, 297 ± 20.3, 170 ± 41.2, and 123 ± 4.2 μg/kg of FB contents were detected in the lungs in the high FB_1_ + DON group, the moderate FB_1_ + DON group, and the low FB_1_ + DON group, respectively. A statistical difference was found between the high FB_1_ + DON group and the low FB_1_ + DON group (*p* < 0.05). Regarding FB residue, a similar difference was found between the high FB_1_ group and the low FB_1_ group, and no significance was detected between the high FB_1_ group and the moderate FB_1_ group. More interestingly, a dose-dependent manner of FB residue was found in the gizzards and in the lungs post-treatment. As for DON residue, no dose effect was found in the lung tissues (Table 4).

**TABLE 4:** Residue of FB/DON in the gizzard and lungs of newborn chickens (μg/kg).

Samples	Groups	FB_1_ + DON	FB_1_	FB_2_	FB_1_ + FB_2_ + FB_3_	DON	CONTROL
Gizzard	High group	260 ± 27.3^a^/10.2 ± 2.2^a^	193 ± 15.9^a^	128 ± 12.7^a^	221 ± 13.0^a^	No data	0
	Moderate group	129 ± 21.0^b^/11 ± 1.8^a^	110 ± 13.9^a^	67 ± 11.0^b^	155 ± 12.3^a^	10 ± 2.8^a^	0
	Low group	98 ± 10.2^c^/3 ± 2.7^b^	69 ± 7.2^b^	45 ± 6.5^b^	110 ± 17.9^b^	No data	0
Lung	High group	297 ± 20.3^a^/9 ± 6.1^a^	216 ± 16.2^a^	202 ± 13.6^a^	253 ± 15.1^a^	No data	0
	Moderate group	170 ± 41.2^a^/7 ± 3.1^a^	131 ± 12.6^a^	83 ± 7.9^b^	162 ± 11.3^a^	3 ± 1.3^b^	0
	Low group	123 ± 4.2^b^/10 ± 2.4^a^	65 ± 10.5^b^	65 ± 1.5^c^	128 ± 12.4^b^	No data	0

*^*a*^*p* > 0.05 when the high FB_1_ + DON group or the high FB_1_ group, or the high FB_2_ group or the FB_1_ + FB_2_ + FB_3_ group was compared to the moderate group and the low groups in the same column.*

*^*b*^*p* < 0.05 when the high FB_1_ + DON group, or the high FB_1_ group, or the high FB_2_ group, or the FB_1_ + FB_2_ + FB_3_ group was compared to the moderate and the low group in the same column.*

*^*c*^*p* < 0.01 when the high FB_1_ + DON group, the high FB_1_ group, the high FB_2_ group, or the FB_1_ + FB_2_ + FB_3_ group was compared to the moderate group and the low group in the same column. The data were expressed as the mean ± SD.*

## Discussion

In our survey, out of 293 feed diets, residues of FB and DON were two dominant mycotoxins both in feedstuff and concentrated poultry diets. More interestingly, high contaminations of FB and DON were found in the egg albumens collected from Southern China (Guangxi and Guangdong provinces) compared to Northern China (Hebei province). Although FB and DON contaminations were lower than the maximal tolerance limits (MTLs) of Chinese feed mycotoxins (Atiqul Haque et al., 2020), the above two egg-borne mycotoxins were equal to 10% of the residual mycotoxins in poultry feeds, indicating higher contamination in comparison with European MTLs (20–100 mg/kg) (Cordeiro et al., 2013). Our investigation indicated that residues of FB and DON could be absorbed into the gastrointestinal system and delivered to breeder eggs and commercial consumable eggs, threatening the development of new hatching birds and human consumers *via* eating eggs or egg products.

In previous reports, FB was the most dominant mycotoxin in maize and sorghum, at 101–1838 μg/kg in maize and 81.5–361 μg/kg in sorghum (Warth et al., 2012). Moreover, the two mycotoxins were common contamination in feedstuffs and poultry diets during the hot season, in comparison with a low-temperature climate. Our data confirm that mycotoxin production correlates with high temperatures (Spanic et al., 2019). In our study, ZEN, OTA, and AFT contaminations were determined to be low residues in both poultry diets and breeder eggs, which was different from high contaminations of OTA and AFT in Pakistan due to diverse climates (Khan et al., 2014).

There are numerous conflicts between feed-borne mycotoxins and layers’ production. Regarding the egg security due to mycotoxin contaminations, limited data have been obtained on the transmission of mycotoxins from feed to eggs and meat tissues. A previous report indicated that roughly 0.31% of DON in feed went into the eggs (Prelusky et al., 1989). Laying hens were fed a diet containing DON [approximately 20 mg/kg (−1)] and ZEN [0.5 mg kg (−1)], and no adverse effects on egg production were observed because the levels of DON were insignificant compared to other sources (Sypecka et al., 2004). Furthermore, low contamination of DON (2.6–17.9 μg/kg) and its metabolite de-epoxy-DON (DOM-1, 2.4–23.7 μg/kg) were detected in home-produced egg samples in Belgium during autumn 2006 and spring 2007, and consumption of these eggs contaminated with DON contributed to less than 1% of the provisional maximum tolerable daily intake of 1000 μg/kg body weight established by FAO/WHO (Tangni et al., 2009). Later on, birds received mycotoxin-contaminated diets for 6 weeks and followed by a 4-week recovery phase. Egg quality, size, and yolk weight were reduced significantly, while egg shape was ameliorated (Lee et al., 2012). More recently, out of 152 egg samples from Jiangsu (JS), Zhejiang (ZJ), and Shanghai (SH) in China, the main mycotoxins were DON, 15-AcDON, and 3-AcDON, with medians of 45.9 μg/kg, 51.5 μg/kg, and 5.94 μg/kg, respectively, while 0.35 μg/kg of ZEN metabolite (β-ZAL) was detected, and AFTB_1_ was found in only one sample at a concentration of 1.46 μg/kg. The aforementioned egg residuals were lower than DON contaminations in our survey due to the mild climate in above three regions in Southern China. Based on a point evaluation and the Monte Carlo model, eggs and chicken tissues contributed a small amount to the mycotoxin intake for children, adults, and elderly adults (Wang et al., 2018). Our survey showed that the residues of FB and DON were much higher than the above reports. The different levels of mycotoxins in eggs might be associated with test methods and sampling seasons. Our samples were collected in the hot season and determined using a commercial ELISA kit. Low-mycotoxin residues were determined in the cold season based on our survey (data not shown). As children are sensitive to mycotoxin levels, it is worthwhile to pay attention to egg security during the hot season, along with egg storage methods.

In our study, a dose-dependent hatching rate was detected in the FB_1_ groups, the FB_2_ groups, and the FB_1_ + DON groups, suggesting a high risk to the developing embryos of breeder eggs. More importantly, both FB1 and DON *via* egg residuals yielded a harmful syngenetic effect on chicken progeny, threatening the sustainable poultry industry. Although FB contaminations are commonly documented in animal feed and feedstuff, the association between FB mycotoxins and hatching rate or gizzard ulcerations has not yet been thoroughly investigated. There are 28 known important FB analogy, among which FB_1_ is the most prominent, followed by FB_2_ and FB_3_. FB_1_ is known as a cancer promoter and plays a significant role in carcinogenesis in humans and immunosuppression in birds. Hatchability was adversely affected by the presence of DON in Lohmann brown (LB) hens’ diet, while the hatchability of Lohmann LSL chicks was significantly higher than that of LB chicks. Moreover, the spleen relative weight and gizzard relative weight were significantly decreased in the hatching chicks post-feeding of the contaminated diets to the hens compared with the control group, indicating a negative impact of DON in LB hens’ diet on fertility and hatchability (Ebrahem et al., 2014). In a previous study, breeder eggs were injected with serial doses of OTA before incubation, and the crown-to-rump length, optic cups, and eye lens diameters were reduced significantly. More interestingly, neural tube closure defects were higher in the OTA-treated embryos. Teratogenic defects and embryonic mortalities were higher in the groups administered high doses of OTA (Zahoor Ul et al., 2011). Prior to incubation, AFB_1_ was injected into the air space of the breeder eggs. Poor embryonic development of the tibial growth plate and skeletal disorders were observed in the hatched broilers during growth (Oznurlu et al., 2010).

Regarding gizzard ulcerations, the epithelium layer of the innermost mucosa is of crucial importance for intestinal barrier functioning. Generally, FB_1_ increases intestinal cell apoptosis, reducing the intestinal barrier and causing immune dysfunction (Gulbahce Mutlu et al., 2018). In the present study, an increase of gizzard ulcerations and hemorrhagic lesions in the lungs were observed in the synergetic high FB_1_ + DON group, the moderate dose of the FB_1_ + DON group, the high dose of the FB_1_ group, and the moderate dose of the FB_1_ group, as compared to the other groups. The above data demonstrated that FB_1_ mycotoxins have a positive effect on gizzard ulceration and lung inflammation. The synergetic combinations of FB1 and DON exacerbated gizzard ulcerations and hatching rate of chicken progenies. A possible mechanism might be associated with the accumulation of two toxins in a long term. Broiler chicks fed FB_1_-contaminated feed showed an altered sphingosine/sphingosine ratio, a biomarker of the FB effect due to disruption of sphingolipid metabolism. FB_1_ shares the same structure as cellular sphingolipids, and sphingolipids are responsible for neurological and immunological diseases, as well as cancer (Grenier et al., 2015). Secondly, FB_1_ altered the integrity of the intestinal barrier and promoted translocation of bacteria by suppressing the tight junction protein expression level. On other hand, DON was found to induce necrotic lesions in the GIT by increasing intestinal permeability and nutrition malabsorption (Liew and Mohd-Redzwan, 2018). In the present study, the combination of FB_1_ and DON induced significant gizzard ulcerations in a dose-dependent manner, indicating synergic effects of the two mycotoxin contaminations in breeder eggs. The GIT is responsible for feed ingestion, digestion, nutrient absorption, and the immune response in newborn chickens. A more comprehensive strategy is urgently required to maintain chicken health and food sustainability.

## Conclusion

Our investigation attempted to highlight how feed-borne mycotoxins were transferred to breeder eggs by checking mycotoxin residues in yolk and albumen samples. Using an embryonated egg model, FB_1_ or the combination of FB_1_ with DON contributed to poor hatching rate and early inflammations of the gizzard ulcerations and hemorrhagic lungs, leading to higher mortality of the progeny of breeder hens and threatening consumable egg safety. Regardless, more studies are needed to elucidate the interaction between mycotoxins and the development of the embryonated chickens, and the implication of such interactions for mycotoxicosis prevention measures.

## Data Availability Statement

The original contributions presented in the study are included in the article/supplementary material, further inquiries can be directed to the corresponding author.

## Ethics Statement

The animal study was reviewed and approved by the China Agricultural University.

## Author Contributions

CH: conceptualization and methodology. YW: data curation and writing—original draft. QL, QS, XL, YW, and HQ: investigation. QS and MH: project administration. YW and QF: resources. CH and QF: writing—review and editing. All authors contributed to the article and approved the submitted version.

## Conflict of Interest

The authors declare that the research was conducted in the absence of any commercial or financial relationships that could be construed as a potential conflict of interest.
